# England’s Mistake in Regard to Army Dentistry

**Published:** 1918-11

**Authors:** 


### ENGLAND'S MISTAKE IN REGARD TO ARMY DENTISTRY

''That the wastage of fighting man-power resulted from lack of proper attention to soldiers' teeth was,'' says a daily paper succinctly, ''the evidence of members of the British Dental Association before the Parliamentary Committee who were appointed to enquire into the subject. They said that a bad condition of the teeth rendered a soldier liable to illness or reduced his chance of recovery in other cases. The committee now recommend that greater attention be paid to the teeth of soldiers while in training in this country—also that there should be more ambulances and that the military dental service should be under the direction of experts.'' There is no commentary to this little *précis* of the latest scene in the long tragi-comedy of dentistry and the war authorities and one has a feeling that the layman who drew it up must have shared in advance the anger and shame of the average reader and decided to let the facts speak for themselves. One can only dimly picture the sentiments of the dental practitioners who, with clear imaginative vision of the long tale of avoidable pain and misery, confined themselves when, at the truly British hour for action, the eleventh hour, they at last got a hearing, to pointing out that it is wasteful to waste.

But some comfort may be drawn from the outlook ahead. It would almost appear that government departments are the last strongholds of dental ignorance; for references to the relationship of teeth to health are now, almost invariably, a prominent feature in articles on hygiene and from the pens of all sorts and conditions of laymen, and women, come, with a greater frequency than ever, articles whose main burden almost amounts to the statement that good teeth are the alpha and omega of good health; and most particularly is it insisted that attention to the teeth of children, early, prompt, regular and continuous attention is the only way to ensure their physical well-being. Look after the teeth, many hygienists seem to proclaim, and the diet, and the health will look after itself. Most dentists it is to be presumed, have held this faith for some long time past. But the difficulty, almost amounting to an impossibility, as long as dentists are private practitioners, of publicly advertising this fact in their own persons, has delayed the growth of public recognition of the truth. But there is another thing that has delayed it and that is the remarkable slowness of the scientific, analytic, relatively inartistic English to perceive the "relationship" of things.

So much for England, cumbered for so long with world-wide possessions and cares. In the compact little republic of Switzerland, self-contained and free to polish in peace its own door-knocker, the problem of dental caries is, if one may judge by the general tenor of the deliberations of a recent meeting of the Swiss Odontological Society, in an advanced state of solution. We read, for instance, that caries of the teeth is to be considered as "a symptom, a constitutional malady due to irrational diet," and that war bread is an undeniable step in advance towards a rational diet—which should be made up of raw, and hard stuffs, masticated with perfect completeness. In Switzerland, then, the battle cry of the dentists is "look after the diet and the teeth will look after themselves." Amongst the reviews of Swiss medical journals is a notice of an article on "Senility of the Tissues of the Teeth in Soldiers." The author of the article apparently claims that the very frequent signs of precocious senility in the jaws and teeth of soldiers—such as excessive mineralization and loss of elasticity in the bony tissue of the jaws—are attributable to fatigue and to a diet too rich in starch. There may be, he considers, other contributory factors, but the two mentioned are, in his opinion, the most important. In all the many references that one meets nowadays in the Swiss dental press to the question of the relationship between diet and teeth, there is to be seen an underlying conviction that the direct cause of dental troubles is to be found in diet and the secondary indirect causes in states not yet sufficiently well elucidated to permit of the working out of an efficient prophylactic treatment. But if the direct cause is clear and can be removed, will not the secondary causes cease to operate? Further, so long as the direct cause remains freely operative, is it not labor misspent to endeavor to trace out possible secondary causes?—British *Dental Record.*

